# Ibuprofen intake after apicoectomy–Quantitative indicator for postoperative pain 

**DOI:** 10.4317/jced.60559

**Published:** 2023-06-01

**Authors:** Ivan-Hristov Arabadzhiev, Carsten Nix

**Affiliations:** 1 Oral Surgeon, Master in Dental Medicine, Master in Public Healthcare and Healthcare Manegement; Oral Surgeon at Oral Surgery Office Dr. Carsten Nix, Landstuhl, Germany; 2 Oral Surgeon, Doctor of dental medicine, Owner of Oral Surgery Office Dr. Carsten Nix, Landstuhl, Germany

## Abstract

**Background:**

Apicoectomy is one of the procedures that are most frequently performed by specialists in oral surgery. This paper presents an analysis of Ibuprofen consumption after apicoectomy and factors such as patient’s age, sex and type of resected tooth. Ibuprofen intake is treated as a quantitative indicator of pain sensation.

**Material and Methods:**

The presented data cover 89 operations with 98 resected teeth. All those apicoectomies were performed by one and the same specialist in oral surgery and all patients were scheduled for a control examination on the day following the intervention. The reported Ibuprofen intake was recorded and analyzed afterwards.

**Results:**

The mean number of consumed Ibuprofen 400 mg tablets, necessary to eliminate the pain was 1.71 (SD±1.33). Gender was not established as responsible for statistically significant differences. Poor negative statistical correlation was established between age and number of consumed tablets. Older patients used a smaller amount of analgesics. The intake after resection of mandibular molars was statistically significantly higher versus that of the other teeth groups. Eighteen of the patients did not consume any analgesic tablets, constituting 18.3% of the whole group. Two patients needed 5 tablets which was the greatest reported intake.

**Conclusions:**

Apicoectomy leads to low Ibuprofen intake. The sex is not a statistically significant factor for Ibuprofen use. Poor negative correlation is observed between age and the amount of administered analgesics. This consumption is increased at resection of mandibular molars compared to that for other teeth groups. Almost one fifth of the patients did not need analgesics during the first postoperative day.

** Key words:**Apicoectomy, postoperative pain, Ibuprofen, oral surgery.

## Introduction

Apicoectomy is one of the most frequently implemented procedures by specialists in oral surgery (1). Numerous publications have revealed that the peak in pain symptomatics was during the first postoperative hours, first night or the following first postoperative day (2-11). Although postoperative pain has been studied many times using visual analogue scale (VAS) and many impacting factors have been analyzed, our study in the Pubmed database did not reveal articles treating the particular quantitative analgesics intake.

Age, sex and localization of the resected tooth are factors predefining the pain extent after apical surgery (9-11). Although those relationships have been outlined in a number of publications and in spite of available measurements with the visual analogue scale (VAS), there is no practical quantitative measurement of those differences reported.

Ibuprofen is a widely administered medicine in oral surgery, reducing effectively postoperative pain (12-14). Ibuprofen safety as pain reducing agent is similar to that of Paracetamol and has been studied in meta analyzes including on children (15). Ibuprofen is a non-steroidal anti-inflammatory drug inhibiting cyclooxygenase converting arachidonic acid to prostaglandin H2, an important mediator of inflammation, pain and body temperature raise. It is one of the most widely used nonsteroidal anti-inflammatory drugs as it is less likely to provoke gastrointestinal and cardiac side reactions than other nonsteroidal anti-inflammatory drugs (16).

The aim of the present manuscript was to analyze the consumption of Ibuprofen after apicoectomy and factors that determined it, such as age, sex and type of resected tooth. The processed data covered 89 interventions with 98 resected teeth.

## Material and Methods

All patients included in the statistics were advised by general dental medicine practitioners to visit our specialized surgical practice for implementation of apical surgery. The apical surgeries themselves were made by one and the same specialist in oral surgery in order to eliminate fluctuations in the results due to different operation techniques and preferences of the individual surgeons. The interventions were conducted not earlier than 24 hours after a comprehensive consultation and signing a written informed consent. The operations were made under local anesthesia with 3 ml Articain with added Adrenalin 1:200 000 (Ultracain® D-S , Septodont, Niederkassel, Germany). Classic “triangular flap” was used for unified access. The access to the upper molars was through 2 flaps and two bone accesses – one buccal and one palatal. The osteotomies were finalized by a spherical bone cutter (Company Komet Dental, Germany). The resection of the apexes itself required the application of bone cutter after Lindemann (Company Komet Dental, Germany). The retrograde preparation of the tooth channels was made with an ultrasonic tip Sonicflex( KaVo, Biberach ,Germany). Hydrogen peroxide solution (3%) was applied to clean the cavities and bone defects, and stop the bleeding. The channel obturation was made with bioceramics with consistence Putty, NeoPutty of the company Avalon Biomed, Houston, TX, USA . All apexes of the relevant tooth were resected during the surgery, i.e. no selective resections of single apexes of multirooted teeth were included.

After the procedures the patients were prescribed 15 Tablets Clindamycin 300 mg (one Tablet 3 times daily) as well as Ibuprofen 400 mg for pain relief, with advice to consume it when necessary in amounts sufficient to eliminate the pain sensation. The recommendation was not to exceed the daily dose of 5 Tablets or a daily dose of 2 000 mg Ibuprofen.

All patients were asked to come for a control examination on the next day, 24 hours after the surgery. They were asked about the pain levels and amount of pain relief drug taken up to that moment. Patients with allergy to Ibuprofen or who, at their individual initiative had used another pain relieving drug were excluded from the statistics. Patients who did not come for control examination we also excluded from the data analysis.

The raw data is presented in Table 1-  Table 11 cont.-1.

**Table 1:**
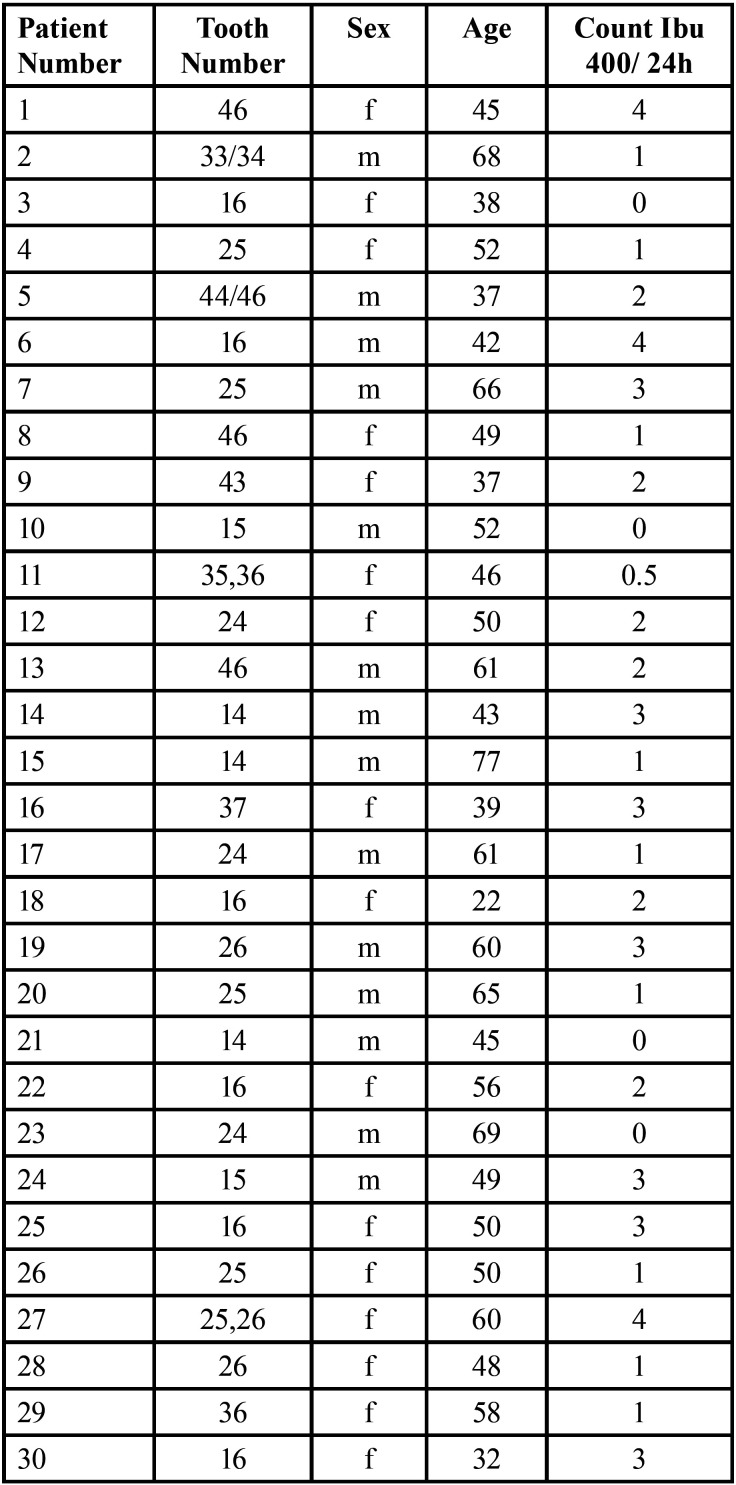
The raw collected data.

**Table 1 cont.**
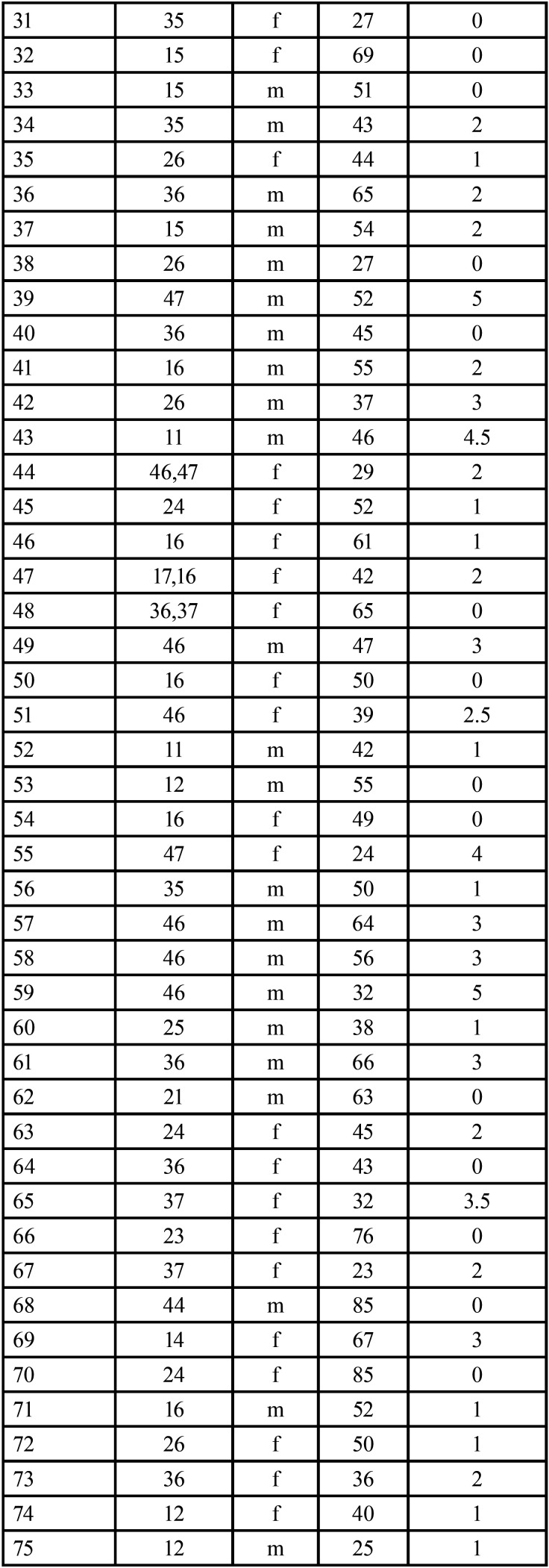
The raw collected data.

**Table 1 cont.-1:**
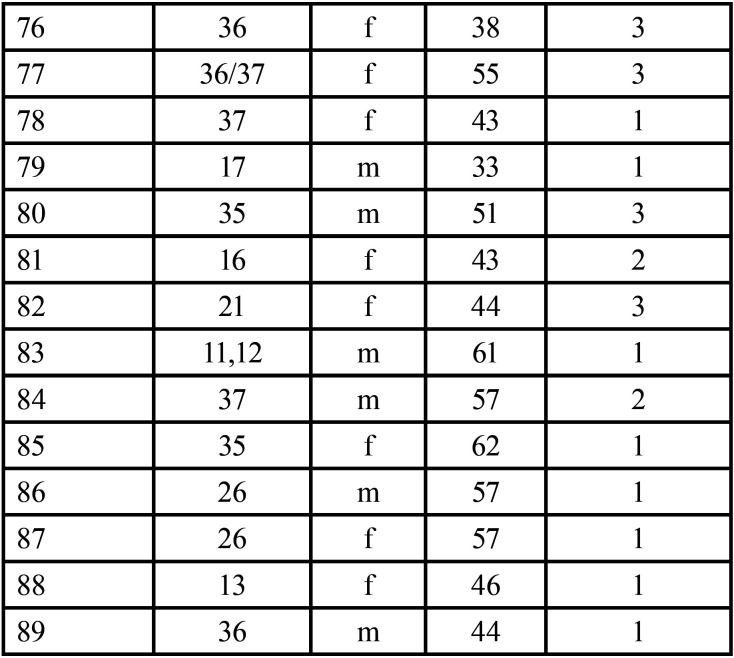
The raw collected data.

## Results

The statistical analysis incorporated the data of 89 patients with 98 resected teeth. Nine of the patients underwent two teeth resections during one visit to the surgeon. The average age of the patients was 49.61 years (SD±13.51). The men/women ratio was 43 to 46.

The mean number of Ibuprofen sufficient to eliminate the pain was 1.71 (SD±1.33). The female group reported 1.6 (SD±1.2) and the male group - 1.76 (SD±1.41).

The distribution by teeth group was as follows: Upper front teeth 1.6 (SD±1.68), Upper premolars 1.25 (SD±1.09), Lower premolars 1.17 (SD±1.07), Upper molars 1.55 (SD±1.16), Lower molars 2.39 (SD±1.35). The patients with two resected teeth during one visit to the specialist formed an individual group extracted from the above six groups. This group reported a consumption of 1.72 (SD±1.18).

The two tailed T-test on the male and female patients’ groups did not reveal a statistically significant difference. The t-value was -0.56535. The *p-value* was 0.573289. The result was not significant at *p* < .05.

The Pearson Correlation Coefficient was calculated for determination of the relationship between consumed Ibuprofen Tablets and patients’ age. The result for Pearson’s r(87)= -0.237, *p*= 0.02897.

The coefficient revealed a poor negative correlation between the age and consumed tablets. Older patients used a smaller number of Tablets. The result could be considered statistically significant at *p*= 0.02897 (at *p* < 0.05)

Eighteen of the patients did not take any analgesic tablet, constituting 18.3% of all patients. Two of the patients reported the greatest number of consumed tablets – 5 Tablets.

## Discussion

There are studies revealing that almost one third of the primary endoscopic therapies might not reach their long term goal (17). One of the possible therapies at primary endodontic failure is endodontic revision, and apicoectomy is another one.

Current techniques and materials for apical surgery enable the improvement of the successful outcome of this procedure. Riis A. *et al*. reported long term results similar to those of endodontic revision (18). Other researchers supported that the surgical approach provided better results than those of conservative therapy. Haxhia E. (2021) (19) reported success percentage rates after endodontic revision of 85% after 6 years, 86.8% after 4 years and 90% after two years. On the other hand, the percentage rates of the group with apical surgery were 88 % after 6 years, 90.5% after four years, and 93.7% after two years. The results showed an advantage of surgical therapy vs. other alternatives. Dioguardi M (20) in a publication in 2022 confirmed those results regarding the two-year results.

Alghamdi F. *et al*. (21) outlined some advantages of apicoectomy with retrograde filling such as reduced period of healing of the periapical lesions compared to classic conservative revision.

Another factor is the possibility for histological examination of the tissues resected by the surgical procedure which is not feasible in the case of endodontic revision. The researchers recommended apical osteotomy as routine procedure before undertaking frontal teeth extraction (22).

The advance in technologies, use of ultrasonic tips and optic magnification contribute to reducing the osteotomy range, pain relief and improve the results of the procedures (22,23). In short term planning apical surgery contributes to faster healing of bone lesions compared to conservative endodontics (24).

Postoperative pain is a major factor for patients’ decision concerning the implementation of surgical treatment. Although there are different opinions on the peak moment of pain sensation after apical resection, most authors have detected it during the first 24 hours after the intervention. Del Fabbro *et al*. (2) supported that the peak moment occurred during the first postoperative day, Christiansen R *et al*. observed a pain maximum during the third postoperative hour (3), and Chong BS and Pitt Ford TR (5) 90% of the patients felt pains from the third to the fifth postoperative hour and 24 hours later the rate fell down to 82%, showing a peak in the first postoperative hours. García B еt al. (6) presenting a literature analysis in 2008 concluded, similar to other researchers that the outlined maximum was felt during the first 24 hours. Tsesis I. *et al*. (8) reported lack of pain for 76.4% of the patients after the first postoperative day.

Those data supported our decision to record the results of our retrospective study one day after the implementation of apical surgery when the peak of pain was expected to have faded away.

The data of our study showed low consumption of Ibuprofen during the first 24 postoperative hours - 1.71 (SD±1.33) Ibuprofen tablets 400 mg. The differences between the male and female patients did not show statistical significance (р>0.05).

The differences by teeth type also did not show significant difference from the average 1,71 for the whole group. The only statistically significant difference was found between the group of resected lower molars and the group of other teeth. For this group the mean consumption of 2.39 (SD±1.35) Ibuprofen Tablets is significantly greater. The two tailed T- Test revealed the value of ‘р’ of .001134.

The group of patients with two adjacent resected teeth (N = 6), though not small, did not show higher levels of analgesics consumption (1.72, SD±1.18) compared to single resections.

Statistically significant effect on Ibuprofen intake was established for the factor “patients’ age”. The analysis of the data showed reduced intake of analgesics with the increase of age. The calculation of the Pearson Correlation Coefficient showed a value of r (87) = -0.237 or derivative of value *p* = 0.02897. Poor, though statistically significant (р<0.05) negative correlation was established.

The group of 18 patients who did not consume any analgesics until the postoperative control examination also turned out to be interesting for us.

The relatively small number of patients (N = 89), as well as the inclusion of only one resection of lower front tooth in the statistics were factors limiting he representativeness of the study.

## Conclusions

Apicoectomy leads to low Ibuprofen intake. The gender is not a statistically significant factor in this aspect. Poor negative correlation is observed between age and amount of consumed analgesics. This intake is increased in case of resection of mandibular molars compared to the other teeth groups. Almost one fifth of the patients did not need any analgesics during the first post operational day.

## References

[1] von Arx T, Roux E, Bürgin W (2014). Treatment decisions in 330 cases referred for apical surgery. J Endod.

[2] Del Fabbro M, Taschieri S, Weinstein R (2009). Quality of life after microscopic periradicular surgery using two different incision techniques: a randomized clinical study. Int Endod J.

[3] Christiansen R, Kirkevang LL, Hørsted-Bindslev P, Wenzel A (2008). Patient discomfort following periapical surgery. Oral Surg Oral Med Oral Pathol Oral Radiol Endod.

[4] Penarrocha M, Garcia B, Marti E, Balaguer J (2006). Pain and inflammation after periapical surgery in 60 patients. J Oral Maxillofac Surg.

[5] Chong BS, Pitt Ford TR (2005). Postoperative pain after root-end resection and filling. Oral Surg Oral Med Oral Pathol Oral Radiol Endod.

[6] García B, Larrazabal C, Peñarrocha M, Peñarrocha M (2008). Pain and swelling in periapical surgery. A literature update. Med Oral Patol Oral Cir Bucal.

[7] Shah SA, Khan I, Shah HS (2011). Effectiveness of submucosal dexamethasone to control postoperative pain < swelling in apicectomy of maxillary anterior teeth. Int J Health Sci (Qassim).

[8] Tsesis I, Fuss Z, Lin S, Tilinger G, Peled M (2003). Analysis of postoperative symptoms following surgical endodontic treatment. Quintessence Int.

[9] Iqbal MK, Kratchman SI, Guess GM, Karabucak B, Kim S (2007). Microscopic periradicular surgery: perioperative predictors for postoperative clinical outcomes and quality of life assessment. J Endod.

[10] Penarrocha M, Garcia B, Marti E, Balaguer J (2006). Pain and inflammation after periapical surgery in 60 patients. J Oral Maxillofac Surg.

[11] Malagise CJ, Khalighinejad N, Patel YT, Jalali P, He J (2021). Severe Pain after Endodontic Surgery: An Analysis of Incidence and Risk Factors. J Endod.

[12] Irvine J, Afrose A, Islam N (2018). Formulation and delivery strategies of ibuprofen: challenges and opportunities. Drug Dev Ind Pharm.

[13] Bahammam MA, Kayal RA, Alasmari DS, Attia MS, Bahammam LA, Hassan MH (2017). Comparison Between Dexamethasone and Ibuprofen for Postoperative Pain Prevention and Control After Surgical Implant Placement: A Double-Masked, Parallel-Group, Placebo-Controlled Randomized Clinical Trial. J Periodontol.

[14] Weiser T, Richter E, Hegewisch A, Muse DD, Lange R (2018). Efficacy and safety of a fixed-dose combination of ibuprofen and caffeine in the management of moderate to severe dental pain after third molar extraction. Eur J Pain.

[15] Kanabar DJ (2017). A clinical and safety review of paracetamol and ibuprofen in children. Inflammopharmacology.

[16] Kim SY, Lee S, Lee Y, Kim H, Kim KM (2021). Effect of single dose preoperative intravenous ibuprofen on postoperative pain and opioid consumption: a systematic review and meta-analysis. Korean J Anesthesiol.

[17] Boucher Y, Matossian L, Rilliard F, Machtou P (2002). Radiographic evaluation of the prevalence and technical quality of root canal treatment in a French subpopulation. Int. Endod. J.

[18] Riis A, Taschieri S, del Fabbro M, Kvist T (2018). Tooth Survival after Surgical or Nonsurgical Endodontic Retreatment: Long-term Follow-up of a Randomized Clinical Trial. J. Endod.

[19] Haxhia E, Ibrahim M, Bhagavatula P (2021). Root-end Surgery or Nonsurgical Retreatment: Are There Differences in Long-term Outcome? J. Endod.

[20] Dioguardi M, Stellacci C, La Femina L, Spirito F, Sovereto D, Laneve E (2022). Comparison of Endodontic Failures between Nonsurgical Retreatment and Endodontic Surgery: Systematic Review and Meta-Analysis with Trial Sequential Analysis. Medicina (Kaunas).

[21] Alghamdi F, Alhaddad AJ, Abuzinadah S (2020). Healing of Periapical Lesions After Surgical Endodontic Retreatment: A Systematic Review. Cureus.

[22] García B, Martorell L, Martí E, Peñarrocha M (2006). Periapical surgery of maxillary posterior teeth. A review of the literature. Med Oral Patol Oral Cir Bucal.

[23] von Arx T, Kurt B (1999). Root-end cavity preparation after apicoectomy using a new type of sonic and diamond- surfaced retrotip: a 1 year follow-up study. J Oral Maxillofac Surg.

[24] Alghamdi F, Alhaddad AJ, Abuzinadah S (2020). Healing of Periapical Lesions After Surgical Endodontic Retreatment: A Systematic Review. Cureus.

